# Burnout, self-rated general health and life satisfaction among teachers and other academic occupational groups

**DOI:** 10.3389/fpubh.2023.1209995

**Published:** 2023-08-11

**Authors:** Till Beutel, Clemens Koestner, Philipp S. Wild, Thomas Münzel, Manfred E. Beutel, Karl J. Lackner, Norbert Pfeiffer, Matthias Nübling, Jan Becker, Stephan Letzel

**Affiliations:** ^1^Institute for Teachers’ Health, University Medical Center of the Johannes Gutenberg University of Mainz, Mainz, Germany; ^2^Institute of Occupational, Social and Environmental Medicine, University Medical Center of the University of Mainz, Mainz, Germany; ^3^Preventive Cardiology and Preventive Medicine, Department of Cardiology, University Medical Center of the Johannes Gutenberg-University Mainz, Mainz, Germany; ^4^Center for Thrombosis and Hemostasis, University Medical Center of the Johannes Gutenberg-University Mainz, Mainz, Germany; ^5^DZHK (German Center for Cardiovascular Research), Partner Site RhineMain, Mainz, Germany; ^6^Institute of Molecular Biology (IMB), Mainz, Germany; ^7^Department of Cardiology – Cardiology I, University Medical Center of the Johannes Gutenberg-University Mainz, Mainz, Germany; ^8^Department of Psychosomatic Medicine and Psychotherapy, University Medical Center of the Johannes Gutenberg-University Mainz, Mainz, Germany; ^9^Institute of Clinical Chemistry and Laboratory Medicine, University Medical Center of the Johannes Gutenberg-University Mainz, Mainz, Germany; ^10^Department of Ophthalmology, University Medical Center of the Johannes Gutenberg-University Mainz, Mainz, Germany; ^11^FFAW: Freiburg Research Centre for Occupational Sciences, Freiburg, Germany

**Keywords:** teachers, work psychology, strains, burnout, occupational group comparison, work-related stress

## Abstract

**Introduction:**

Teachers work in a job with specific demands that can strain individual coping capabilities and can pose a risk for the development of psychological problems. Prior studies showed that teachers – in comparison with other occupational groups – had high risks of job-related psychological exhaustion. In our study we compared teachers and other occupational groups on burnout, general life satisfaction and self-rated general health. In addition, we analyzed if sociodemographic and job-related factors were relevant predictors of these outcomes.

**Methods:**

We analyzed data from a total of 1,500 subjects arising from the Gutenberg Health Study. Binary logistic regression models and descriptive statistics were calculated to determine potential differences between the occupational group membership and the predictive values of sociodemographic and job-related variables.

**Results:**

The occupational groups did not differ significantly in terms of burnout, self-rated general health and satisfaction with life. Logistic regression models showed which sociodemographic and job-related variables were associated with the outcomes. Female sex, part-time employment as well as work-privacy conflicts showed particular predictive relevance.

**Discussion:**

Job-related interventions for teachers should aim at specific strains, e.g., arising out of work-privacy conflicts where interventions should focus on support of female teachers.

## 1. Introduction

Various studies have emphasized that psychological stress plays a major role for teachers in their daily work (1–4). Particular stress factors in teachers can include: dealing with challenging students’ or parents’ behavior as well as student diversity with correspondingly high social-communicative demands; discrepant role expectations; high density of social interactions; conflicts or lack of supervisory support; few opportunities for breaks and regeneration during the time spent at school; mixing of work and leisure time as well as physical stressors from the work environment [e.g., noise (3, 5, 6)]. Taken together, these and other factors form a structure of demands or stress that strain individual coping capabilities and can pose a potential risk for the development of psychological problems in teachers (7, 8). Stress can be interpreted as the whole of all influences (e.g., physical, biological, chemical, and mental or social) that can induce reactions from an organism. Strains (e.g., physical, psychological, and behavioral) resulting from the impact of stress depend on the individual characteristics (e.g., sociodemographic and biographical) of a person (9, 10).

Stress and strains in teachers have been intensively researched in the recent years and decades (3). Job-related stress and job dissatisfaction in teachers are associated with risk for the development of burnout (11). Some differences in stress and strains seem to be associated with sociodemographic variables. Female teachers reported more job-related stress and emotional stress (5, 12) and higher levels of emotional exhaustion (5). Another study revealed, that symptoms of exhaustion were twice as common in female (21%) than in male (11%) teachers (13). Another study detected higher psychological fatigue scores in female teachers as well (14). Absenteeism rates in female teachers were also higher than in male teachers (12). These gender differences might be caused by different roles. One assumption is, that women might be more strained due to private responsibilities, e.g., caretaking or household responsibilities (15). Other studies point out that there are age-related differences in psychological strains such as burnout in educational staff; the highest incidence of burnout was found in teachers within 50–59 years of age (6.6%) and the lowest within 18–29 years of age (1.4%) (16). More subgroup differences regarding burnout in teachers could be observed, e.g., when stratified by levels of teaching, e.g., elementary school teachers showed higher burnout scores compared to higher education teachers (17). Teachers with eight or more years of experience scored higher on emotional exhaustion compared to teachers in their first years of work (18).

To determine job-related stress, one approach is to compare different occupational groups with each other (19), taking job-related stress variables into account as well as outcome variables. One example of this approach was performed in a study conducted by Nübling et al. (20). Associations between occupational groups and a multitude of work and health related variables were analyzed. Variance in burnout (η^2^ = 0.19), satisfaction with life (η^2^ = 0.15) and general health (η^2^ = 0.16) was explained by the affiliation to different occupational groups.

Out of nine different occupational groups (21), teachers and social workers – compared to other occupational groups – most often showed effort-reward imbalances as well as effort tendencies in the sense of the effort-reward imbalance model (22). A representative survey of more than 30,000 employees from 67 occupational groups (23) provided another insight into job-related psychological stress. The comparison of occupational groups showed that teachers from different types of schools had the highest risk of job-related psychological exhaustion (odds ratios for teachers from different types of schools between 1.9 and 3.4). Physicians on the other hand showed lower levels of psychological exhaustion. In terms of workload and work pressure, however, teachers and physicians showed comparable results. Teachers – with the same reported workload – had significantly higher scores for psychological exhaustion than individuals from other occupational groups (23). Teachers also showed slightly higher average burnout levels than other occupational groups (24). However, a review showed that – compared to other occupational groups – teachers were less likely to suffer from psychological impairment or disorders, while low educational attainment was more likely to be associated with mental health problems (25). These results are also supported by other studies (26) and can partly be explained by the fact that employees with low education often work in high-strain jobs (25).

With regard to **work-privacy conflicts**, teachers on average, reported significantly higher levels of stress than the mean of other occupational groups (24, 27). The spatial overlap of work and private life at two workplaces (school & home) is an additional structural characteristic of school teachers (3). It is therefore not difficult to imagine how conflicts between work and privacy could arise from this.

Teachers also showed lower values for **role clarity** compared to all occupational groups found in a previous study. In particular, younger teachers showed unfavorable scores on this variable, with role clarity increasing with years of service (24). Compared to the general population, teachers (especially female teachers) not only showed worse psychological but also perceived their physical health status worse (12).Contrary to that, it has been shown that the objective health status (e.g., cardiovascular risk factors) and also the health behavior in teachers was better than in the general population (28). Along the same, another study showed that the health behavior of teachers was better compared to other occupational groups (29).

Other job-related factors were also more positive in teachers. The prevalence of **bullying** was lower in teachers compared to other occupational groups. Further, resources should be taken into account as well. In comparison with other occupational groups, **importance of work** as well as the **commitment to the workplace** were rated slightly better by teachers (24).

As shown above, some differences in strains seem to derive from the affiliation to a certain occupational group, others can be explained by sociodemographic or work-related variables within occupational groups.

The aims of our study are to investigate the following issues:

1. Do sociodemographic & job-related variables in school teachers differ from other academic occupational groups?
2. Do teachers differ from other occupational groups regarding the outcome variables burnout, general life satisfaction, and self-rated general health?
3. Which of the sociodemographic variables and job-related variables are particularly relevant in predicting the outcome variables?

## 2. Materials and methods

### 2.1. Sample

The sample analyzed for the present study was drawn from the data set of the Gutenberg Health Study (GHS) (30). The GHS is a large-scale, prospective, and representative population study conducted since 2007. The aim of the study is to identify risk factors and causes of the most common diseases. With over 15,000 participants from the Rhine-Main region, it is one of the largest regional health studies in the world.

Data on mental health related variables were collected by questionnaires during the study days. Participation in the study was voluntary and the subjects were able to withdraw from the study at any time. In the present study data-collection took place between 2007–2017 with a total of 1,500 participants (37.5% female, age *M* = 48, SD = 8.2) was used. Inclusion criterion was the affiliation to one of the following occupational groups:

- school teachers (*n* = 126, 69.8% female, age *M* = 51.2, SD = 8.4),
- teaching and training professions (non-school teachers, e.g., in-house education and training or professors at universities) (*n* = 66, 45.5% female, age *M* = 48.8, SD = 8.7),
- medical doctors and dentists (*n* = 44, 36.4% female, age *M* = 51.2, SD = 9.1), and
- other academic professions (*n* = 1,264, 33.9% female, age *M* = 47.5, SD = 8.1).

The group allocation was based on the occupational data recorded as part of the GHS. These were available in the form of codes for the classification of occupations (Klassifikation der Berufe: KLDB) used by the German Federal Employment Agency. The reference group “other academics” comprised of persons with educational degrees from a “university of applied sciences” or “university.” In order to avoid autocorrelations – and thus an overestimation of the strength of the associations under consideration – individuals already assigned to occupational groups (a) to (c) were not additionally assigned to reference group (d).

On the one hand**, school teachers (ST)** were compared to academically graduated participants who worked as **teaching and training professionals (TT)**. This occupational group was chosen because of the occupational proximity due to the work content. In addition, school teachers were compared to another academic occupation with high levels of personal interactions, but with different work content. This occupational group for comparison was **medical doctors and dentists (MD)**. The broader group of **other academics (AC)** was selected as the reference group for all intergroup comparisons because of a comparable socioeconomic status.

### 2.2. Measurements

The independent variables of interest are listed below this paragraph. The scales to measure the work-related variables (h-l, see below) were part of the Copenhagen Psychosocial Questionnaire (COPSOQ) (31, 32). The COPSOQ is based on several theoretical models (e. g., Demand-Control Support Model or Effort-Reward-Imbalance Model) and is a scientifically validated tool, commonly employed in psychosocial risk assessment at workplaces (31). This comprehensive questionnaire, originally developed in Denmark (32) evaluates several psychosocial factors including demands at work, work organization, interpersonal relations, work-individual interface, values at the workplace, health, and well-being. Through detailed inquiry, the COPSOQ identifies areas for potential psychosocial risk intervention, facilitating healthier work environments. Each item was answered on a Likert scale. The scales used for the present study are described below.

#### 2.2.1. Independent variables

**Sociodemographic variables**

- Sex (men/women)
- Age
- SES

**Occupational groups**

- School teachers
- Teaching and training professions
- Medical doctors and dentists
- Other academic professions

**Work-related variables**

- Work-privacy conflicts (5 items, 5-point Likert scale, sample item: “The demands of my work interfere with my personal and family life”)
- Role clarity (3 items, 5-point Likert, scale, sample item: *“Are there clear goals for your work?”*)
- Meaning of work (2 items, 5-point Likert, scale, sample item: *“Do you feel that your work is important?”*)
- Bullying (1 item, 5-point Likert, scale: “Do you often feel unfairly criticized, bullied, or embarrassed in front of others by colleagues and supervisors?”)
- Satisfaction with work (7 items, 4-point Likert scale, sample item: “Looking at your work situation overall, how satisfied are you with your job overall, considering all circumstances?”)
- Work schedule (1 item, part-time vs. full-time)

#### 2.2.2. Dependent variables

- Burnout (6 items, sample item: “How often do you feel drained?”)
- General life satisfaction (5 items, sample item: “My living conditions are excellent.”)
- Self-rated general health (1 item: “If you rate the best conceivable state of health as 10 points and the worst conceivable state of health as 0 points: How many points do you then assign to your current state of health?”)

### 2.3. Ethics approval

The authors confirm that the study was approved by the local ethics committee [ethics committee of the state medical association RLP; ethics committee vote: 837.020.07(5555)] and was performed in accordance with the ethical standards as laid down in the 1964 declaration of Helsinki and its later amendments. Written informed consent was obtained from all participants.

### 2.4. Statistical analyses/evaluation

Descriptive data (means, relative frequencies, and standard deviations) were calculated for the variables used in the analyses, both for the total sample and for each individual occupational group. In the case of particularly skewed distributions of scales (skewness >1), the median and interquartile range were calculated rather than the scale mean. In the data analyses, the three occupational groups (a) school teachers, (b) teaching and training professions, and (c) medical doctors and dentists were considered separately.

The dependent and independent variables (COPSOQ scales, 5-level Likert format) were transformed to values between 0 and 100 for data analyses (transformation for, e.g., 5-level Likert-scales was 1 = 100, 2 = 75, 3 = 50, 4 = 25, and 5 = 0 and accordingly for different numbers of Likert-scale levels). High values represent high expressions of the construct examined in the respective scale.

Binary logistic regression models (Poisson regression) were calculated in order to estimate the predictive value of sociodemographic variables, the three compared occupational groups, and the aforementioned independent variables on the dependent variables. The reference group for logistic regression analyses regarding the occupational groups was the occupational group of other academic professions.

For each dependent variable, three separate regression models were set up with different levels of adjustment; the variables considered in a previous level were retained in the next level (see Table 1). In the logistic regression models (Poisson regression), a median split was performed for each of the strains (dependent variables) to dichotomize values below or above 50. In the fully specified regression model (third regression model) the dependent variables of the different occupational groups were adjusted for sex, age, socio-economic status (SES^1^), working time model, and for the scales satisfaction with work, work-privacy conflicts, role clarity, meaning of work, and bullying. To enable a more precise differentiation, all analyses were carried out separately for female and male participants.

**Table 1:** Levels of regression models and their respective content specification.

Levels of adjustment	Adjusted for
1. Regression model	Age Sex female male SES Working time model full-time part-time
2. Regression model	Occupational group classification school teachers teaching and training professions medical doctors and dentists other academics
3. Regression model	Job-related factors job satisfaction work-privacy conflicts role clarity meaning of work bullying

## 3. Results

Descriptive results are shown below in Table 2.

**Table 2:** Descriptive statistics.

Variable	Total (*N* = 1500)	School teachers (8.4%, *N* = 126)	Teaching and training professions (4.4%, *N* = 66)	Medical doctors and dentists (2.9%, *N* = 44)	Other academics (84.3%, *N* = 1,264)
Socio-demographic variables					
Sex (Women)	37.5% (*N* = 562)	69.8% (*N* = 88)	45.5% (*N* = 30)	36.4% (*N* = 16)	33.9% (*N* = 428)
Age (*y*)^±^	48.0 (8.2)	51.2 (8.4)	48.8 (8.7)	51.2 (9.1)	47.5 (8.1)
SES^†, ±^	18.4 (2.0)	19.0 (1.7)	18.7 (2.0)	19.2 (1.7)	18.3 (2.0)
Part-time working (yes)	16.3% (*N* = 244)	27.8% (*N* = 35)	18.2% (*N* = 12)	6.8% (*N* = 3)	15.3% (*N* = 194)
Independent variables					
Bullying ^MD^	0 (0/25.00)	0 (0/25.00)	0 (0/8.33)	2.4 (0/8.09)	3.0 (0/25.00)
Bullying > = 50 (yes)	10.5% (*N* = 157)	9.5% (*N* = 12)	7.6% (*N* = 5)	6.8% (*N* = 3)	10.8% (*N* = 137)
Work-privacy conflict^±^	42.7 (26.51)	49.1 (24.38)	41.6 (25.63)	56.3 (26.31)	41.6 (26.57)
Work-privacy conflict> = 50 (yes)	43.4% (*N* = 651)	48.4% (*N* = 61)	43.9% (*N* = 29)	70.5% (*N* = 31)	41.9% (*N* = 530)
Clarity of roles-scale^±^	80.1 (15.87)	80.2 (15.11)	81.3 (15.33)	89.1 (12.58)	79.7 (15.99)
Clarity of roles-scale> = 50 (yes)	96.9% (*N* = 1,454)	96.8% (*N* = 122)	97.0% (*N* = 64)	100.0% (*N* = 44)	96.8% (*N* = 1,224)
Meaning of work-scale^±^	78.5 (17.55)	83.5 (14.85)	88.3 (13.98)	90.7 (12.48)	77.0 (17.70)
Meaning of work-scale> = 50 (yes)	96.5% (*N* = 1,447)	99.2% (*N* = 125)	100.0% (*N* = 66)	100.0% (*N* = 44)	95.9% (*N* = 1,212)
Satisfaction with work-scale^±^	70.3 (14.6)	71.0 (14.0)	75.6 (14.0)	75.1 (13.3)	69.8 (14.6)
Satisfaction with work-scale> = 50 (yes)	92.3% (*N* = 1,385)	94.4% (*N* = 119)	95.5% (*N* = 63)	95.5% (*N* = 42)	91.9% (*N* = 1,161)
Dependent variables					
Personal burnout-scale^±^	36.4 (17.19)	41.1 (17.41)	37.4 (17.64)	38.7 (16.63)	35.8 (17.10)
Personal burnout-scale> = 50 (yes)	25.2% (*N* = 370/1470)	32.3% (*N* = 40/124)	27.7% (*N* = 18/65)	29.5% (*N* = 13/44)	24.2% (*N* = 299/1237)
Satisfaction with life-scale^±^	72.5 (17.32)	75.4 (16.64)	74.7 (15.81)	79.3 (15.56)	71.9 (17.45)
Satisfaction with life-scale> = 50 (yes)	89.5% (*N* = 1318/1473)	92.7% (*N* = 115/124)	90.8% (*N* = 59/65)	93.0% (*N* = 40/43)	89.0% (*N* = 1104/1241)
Self-rated health-scale^MD^	80.0 (70.0/80.0)	80.0 (70.0/80.0)	80.0 (70.0/80.0)	80.0 (70.0/90.0)	80.0 (70.0/80.0)
Self-rated health-scale> = 50 (yes)	94.2% (*N* = 1382/1467)	92.6% (*N* = 112/121)	95.5% (*N* = 63/66)	97.7% (*N* = 43/44)	94.2% (*N* = 1164/1236)

For the results of the scales from the COPSOQ, both the scale mean values (range: 0–100) and the proportion of participants (in % and absolute numbers) with values > =50 (dichotomized variables via median split) are shown. In the case of particularly skewed distributions of individual scales (skewness > 1), not the scale mean values but the respective median and the interquartile range were given. ^†^SES, socioeconomic status. ^±^Mean (SD). ^MD^Median (interquartile range).

The results showed that the proportion of females in the occupational group of school teachers was higher than in the other two occupational groups, namely the teaching and training professions and medical doctors and dentists and it was higher than in the reference group of other academics too. The higher proportion of female school teachers is in line with other surveys of school teachers (e.g., 71.4% female teachers in Rhineland-Palatinate, Germany in 2017/18; (6)). The average age was comparable across the occupational groups, at approximately 50 years. The proportion of part-time employees in the occupational group of school teachers was noticeably higher than in the other two occupational groups and also higher than in the reference group. Regarding the working time model, it is noteworthy that 36.4% of the female school teachers were employed part-time (37.9% in the total sample), whereas only 7.9% of male school teachers were employed part-time (3.3% in the total sample).

### 3.1. Differences in job-related factors

With regard to the job-related variables, the following differences were found between the occupational groups (see Table 2). On the scale **satisfaction with work** the mean value of school teachers was below that of the two comparison groups; however, all three occupational groups were above the mean value of the reference group of other academics.

On the **work-privacy conflicts** scale, the three occupational groups had higher mean values than the reference group of other academics. This was most pronounced in the occupational group of medical doctors and dentists, followed by school teachers.

The highest mean value on the **role clarity** scale was also shown by medical doctors and dentists, while the other groups were at a comparable and lower level.

Regarding the **meaning of work**, the mean value of the school teachers was below that of the two comparison groups, but it was also above the mean value of the reference group of academics.

With regard to **bullying**, all occupational groups and the reference group showed left-skewed (towards zero) distributions. The value of the median for the school teachers was 0, the same value as in the occupational group teaching and training professions. The median for the occupational group of medical doctors and dentists, and the reference group of other academics was only slightly higher. Considering the scaling from 0–100, it can be stated that low values for bullying were found across all occupational groups.

### 3.2. Differences regarding strains

#### 3.2.1. Burnout

For the **burnout** scale, the highest unadjusted mean value was found in the occupational group of teachers, while the two other occupational groups were at a somewhat lower and comparable level. All three occupational groups had higher scale mean values than the reference group of other academics. Dichotomized into values below 50 and values above 50, more female participants (34.1%) than male participants (19.9%) indicated values above 50 across all occupational groups (*N* = 1,470).

Further, occupational group differences regarding the dependent variables were investigated by regression analyses. For this purpose, the regression models were adjusted according to the analysis plan (see Table 1).

In the three occupational groups compared, the mean values in the third logistic regression model for the **burnout** variable were slightly above the mean value of the reference group. Nevertheless, in the regression model, none of the occupational groups considered were significant predictors of burnout (*p* > 0.05).

In the first logistic regression model (variables: sex, age, SES, and working hours model), being female (prevalence ratio (PR)_sex = women_ = 1.59, *p* = <0.0001) was a significant predictor, i.e., high levels of burnout (burnout scores >50) were approximately 1.6 times more likely in female subjects than in male subjects. In the second logistic regression model (additional variable: occupational groups), none of the occupational groups were significant predictors for burnout. In the third logistic regression model (additional variables: satisfaction with work, work-privacy conflicts, role clarity, meaning of work, and bullying) the occupational group of school teachers was equally associated with burnout symptoms compared to the other occupational groups. Regardless of whether the job-related factors were included in the regression analysis, occupational group was not a significant predictor for burnout in the regression model.

The following predictors were significant in terms of increased likelihood of burnout in the third logistic regression model: **female sex** (PR _sex = women_ = 1.44, *p* = <0.001), **part-time working** (PR _part-time working = yes_ = 1.69, *p* = <0.0001), **work-privacy conflicts** (PR = 1.23, *p* = <0.0001), **bullying** (PR = 1.06, *p* = <0.05), and **satisfaction with work** (PR = 0.83, *p* = <0.0001). Full results for the third regression model are presented in Table 3.

**Table 3:** Log-Lin-Poisson regression model 3: burnout.

Effect	Estimate	95% CI		*p*
		*LL*	*UL*	
Fixed effects				
Intercept	0.1321	0.1107	0.1577	**<0.0001**
Sex (women)	1.4419	1.1646	1.7852	**0.00078**
Age (10y)	1.0205	0.9066	1.1487	0.74
SES	0.9634	0.9250	1.0034	0.073
Part-time working	1.6354	1.3049	2.0498	**<0.0001**
Teachers	1.0272	0.7739	1.3633	0.85
Teaching and training professions	1.4003	0.8703	2.2528	0.17
Medical doctors and dentists	0.9611	0.4920	1.8776	0.91
Mobbing (10%)	1.0600	1.0130	1.1092	**0.012**
Work-privacy conflict (10%)	1.2324	1.1849	1.2818	**<0.0001**
Clarity of roles-scale (10%)	1.0589	0.9939	1.1282	0.076
Meaning of work-scale (10%)	0.9583	0.9034	1.0165	0.16
Satisfaction with work-scale (10%)	0.8267	0.7664	0.8919	**<0.0001**

Total *N* = 1,183. CI, confidence interval; LL, lower limit; UL, upper limit. Bold *p*-values are significant at a level of *p* < 0.05.

When evaluated separately for female and male participants, **work-privacy conflicts** were a significant predictor for burnout in both sexes. The following sex difference was found for **working part-time**. Only in female participants was working part-time (PR = 1.69; *p* < 0.0001) a significant predictor in the logistic regression model, not in males. This means that controlling for all variables considered, females who worked part-time were 1.69 times more likely to report burnout scores >50.

#### 3.2.2. Self-rated general health

The results of the **self-rated general health** scale for all occupational groups were skewed to the right (towards 100), therefore the median is reported here instead of the mean. The median was 80.0 for all groups. Dichotomized into values below and above 50, female (93.5%) and male participants (94.7%) reported values above 50 with a comparable frequency across all occupational groups (*N* = 1,467).

The mean values of the variable **self-rated general health** for participants in the occupational groups ST and TT were at a comparable level to those of the reference group of other academics. The self-rated general health of the occupational group MD was above the average of the compared groups.

In the first logistic regression model, age was a significant predictor of self-rated general health (PR_age_ = 0.97, *p* = <0.0001). Participants’ self-rated general health decreased slightly with increasing age. In the second logistic regression model, occupational group was no significant predictor for self-rated general health. Also, in the second logistic regression model (PR_age_ = 0.97, *p* = <0.0001) and in the third logistic regression model (PR_age_ = 0.95, *p* = <0.0001), there was a significantly increased probability for lower values of self-rated general health for older participants, respectively. Occupational group was no significant predictor in any of the regression models. Significant predictors in terms of increased likelihood of self-rated general health scores >50 in the third logistic regression model were: **age** (PR _age_ = 0.95, *p* = <0.0001), **work-privacy conflicts** (PR = 0.99, *p* = <0.0067) and **satisfaction with work** (PR = 1.02, *p* = <0.01). Full results for the third regression model are presented in Table 4.

**Table 4:** Log-Lin-Poisson regression model 3: self-rated health.

Effect	Estimate	95% CI		*p*
		*LL*	*UL*	
Fixed effects				
Intercept	0.9560	0.9378	0.9745	**<0.0001**
Sex (women)	0.9869	0.9532	1.0217	0.46
Age (10y)	0.9543	0.9347	0.9743	**<0.0001**
SES	1.0051	0.9972	1.0131	0.21
Part-time working	0.9980	0.9543	1.0438	0.93
Teachers	1.0135	0.9644	1.0650	0.60
Teaching and training professions	1.0126	0.9574	1.0711	0.66
Medical doctors and dentists	1.0006	0.9177	1.0911	0.99
Mobbing (10%)	0.9912	0.9822	1.0002	0.056
Work-privacy conflict (10%)	0.9915	0.9854	0.9976	**0.0067**
Clarity of roles-scale (10%)	1.0021	0.9906	1.0136	0.73
Meaning of work-scale (10%)	1.0001	0.9879	1.0124	0.99
Satisfaction with work-scale (10%)	1.0170	1.0039	1.0302	**0.011**

Total *N* = 1,188. CI, confidence interval; LL, lower limit; UL, upper limit. Bold *p*-values are significant at a level of *p* < 0.05.

#### 3.2.3. Satisfaction with life

On the **satisfaction with life** scale (unadjusted), the occupational group of medical doctors and dentists showed the highest mean value. The occupational group of teachers as well as those working in teaching and training were somewhat lower in terms of life satisfaction.

The values for the variable **satisfaction with life** were lowest among participants in the reference group of other academics. For the three compared occupational groups TT had the lowest values. The mean value for ST was slightly higher, and the highest satisfaction with life values were found among MD, although the confidence interval was particularly wide.

Female participants (M = 72.1, SD = 18.1, *n* = 562) and male participants (M = 72.8, SD = 16.9, *n* = 938) from all occupational groups showed no significant difference in their mean life satisfaction values, with *t* (1498) = 0.76, *p* = 0.45.

In the first logistic regression model, the SES was a significant predictor of satisfaction with life (PR_SES_ = 1.04, *p* = <0.0001). The same pattern emerged in the second logistic regression model (PR_SES_ = 1.03, *p* = <0.0001) and in the third logistic regression model (PR_SES_ = 1.03, *p* = <0.0001). In each case, SES was a significant predictor for satisfaction with life. Occupational group was no significant predictor in any of the regression models. Significant predictors in the fully adjusted third regression model for satisfaction with life were: higher **SES** (*p* < 0.01), higher **satisfaction with work** (*p* < 0.01), higher **meaning of work** (*p* < 0.01) and less **work-privacy conflicts** (*p* < 0.05). Please refer to Table 5 for the complete results.

**Table 5:** Log-Lin-Poisson regression model 3: satisfaction with life.

Effect	Estimate	95% CI		*p*
		*LL*	*UL*	
Fixed effects				
Intercept	0.9137	0.8881	0.9400	**<0.0001**
Sex (women)	0.9670	0.9226	1.0134	0.16
Age (10y)	0.9816	0.9583	1.0054	0.13
SES	1.0311	1.0177	1.0446	**<0.0001**
Part-time working	1.0283	0.9677	1.0928	0.37
Teachers	1.0257	0.9700	1.0846	0.37
Teaching and training professions	0.9823	0.9090	1.0615	0.65
Medical doctors and dentists	1.0407	0.9916	1.0922	0.11
Mobbing (10%)	0.9867	0.9733	1.0004	0.058
Work-privacy conflict (10%)	0.9905	0.9821	0.9990	**0.028**
Clarity of roles-scale (10%)	0.9857	0.9704	1.0012	0.070
Meaning of work-scale (10%)	1.0251	1.0069	1.0437	**0.0068**
Satisfaction with work-scale (10%)	1.0376	1.0192	1.0562	**<0.0001**

Total *N* = 1,185. CI, confidence interval; LL, lower limit; UL, upper limit. Bold *p*-values are significant at a level of *p* < 0.05.

## 4. Discussion

The present study aimed to examine whether job-related factors and outcome variables (burnout, general health and life satisfaction) differed between school teachers and other academic occupational groups. We further wanted to determine out which of the analyzed job-related variables were particularly relevant in predicting the aforementioned outcome variables.

### 4.1. Job-related factors

With regard to satisfaction with work, all three compared occupational groups (including school teachers) were above the level of the reference group of other academics. School teachers were slightly less satisfied with work compared to teaching and training professions, as well as medical doctors. However, in another study primary teachers showed higher scores on job satisfaction compared to social workers. We further analyzed differences in terms of work privacy conflicts. Again, all three occupational groups showed higher values than the reference group. The overlap of work and private life at two workplaces has been discussed in terms of higher conflicts in teachers (3, 24). However, we did not see pronounced differences in teachers. Instead, the biggest differences were observed in medical doctors. These had the highest and correspondingly least favorable values in this regard. In contrast, role clarity was highest and thus most pronounced among medical doctors and dentists; the other two occupational groups were at comparable levels slightly above the mean of the reference group. In contrast to our results, in another study (24) school teachers rated role clarity worse than individuals of other occupational groups. Differences between the occupational groups were rather minimal in terms of role clarity overall, and can be seen as well-defined. The same was evident in terms of how those occupational groups rated the meaning of their work. Differences were small, with the highest values observed in medical doctors. We also noted that bullying was very little to not at all prevalent within the occupational groups compared.

These results give a first hint towards the possibility that the occupational groups analyzed might have been more similar than expected. Along the same line, it is plausible to assume that the compared occupational groups might be more heterogeneous within each group than expected. We think that the variance for job-related factors in each group outweighed the variance between groups, which led to non-significant predictive values for occupational groups. One explanation might be the narrow selection (academics only) of occupational groups in our study. Studies with more accentuated between-groups differences compared a much wider range of occupational groups (23).

### 4.2. Strains

#### 4.2.1. Burnout

We only detected minimal differences between the considered occupational groups in terms of burnout symptoms.

However, two other studies with large samples of school teachers reported higher burnout scores in teachers compared to the average respondent from other occupational groups (24, 27). This might result from differences in the samples. Our analyzed sample seems to be more homogenous (consisting only of academic professions). None of the three occupational groups were significant predictors for burnout. We again assume that occupational in-group variance outweighs between-group variance.

Nonetheless, other studies (24, 27) provide a useful opportunity for comparison, as the same items were used to measure burnout. This is important since burnout is an indistinct construct (33). Because different definitions and survey instruments of burnout exist, prevalence rates among school teachers vary considerably (34, 35) and results therefore are oftentimes incomparable. A complete burnout syndrome was identified for 1%–5% of female school teachers, and at least one-third of the school teachers showed some symptoms of it (36). A comparison of our teacher sample with the other samples mentioned above showed slightly lower burnout values [our sample: *M* = 41 vs. *M* = 49 (24) or *M* = 46 (27)]. These are rather small differences, which can result from different survey methods. Both mentioned studies recruited large samples of teachers from different schools with a complete survey of a certain school, partly integrated in a risk management. Teachers from our study were random parts of the GHS study population and the sample size of teachers in our study was much smaller. Besides that, differences might also derive from different German states where recruitments took place. In sum, we assume random reasons for the small differences as structural conditions of teachers being compared in these studies are largely similar.

Yet, burnout can be seen as a multi-layered risk factor in the development of mental disorders such as depression (33). To determine factors associated with burnout we computed regression analyses to detect relevant variables for the prediction of burnout symptoms. Female gender was a significant predictor for higher burnout. Another study also found sex differences in the sense that women reported higher burnout scores (24). In this study, women also had less favorable scores on cognitive stress symptoms. Similar results were reported in a Belgian study, in which female teachers had the highest scores on psychological fatigue (14). It is also known from the general population that significant sex differences exist with regard to the subjective experience of stress. For example, various studies have shown that women report a higher number of psychosocial stressors or have more severe stress experiences compared to men (37, 38).

Apart from differences arising from the analyzed occupational groups, our interest was to determine which of the job-related characteristics showed a connection to burnout. Work-privacy conflicts were most relevant in this regard. High conflicts went along with pronounced burnout symptoms in the adjusted regression model. This is in line with similar studies (24, 27), where a linear correlation between work-privacy conflicts and burnout scores has been shown. A reduction of 10 percentage points on the work-privacy conflicts scale was accompanied by an improvement of 4.5 points in burnout scores (24).

Working part-time was also associated with burnout in our study, but interestingly only in female, not in male participants. It is possible that women in part-time work models may not benefit from a reduction in weekly working hours to the same extent as men do. This might be due to traditional gender roles. Women might spend more time on childcare or household duties when reducing working hours and thus experience less of a relief, if there is a relief at all. Higher workloads in women due to higher household responsibilities as a reason for sex differences in terms of fatigue or other demands have been discussed long ago (15). As expected, a higher job satisfaction was associated with lower burnout symptoms.

#### 4.2.2. Self-rated health

Self-rated health is a well-established indicator for health status used in social and health studies. A recent study pointed out the strong association with the biological condition (39). We were interested in differences between the occupational groups as well as associations between job related factors and health status. None of the three occupational groups were significant predictors for self-rated-health. Given the assumption that exclusively academic occupational groups might have comparable socioeconomic status and health education, they therefore might differ only slightly in self-rated general health as an outcome.

When compared to more heterogeneous groups, i.e., the general population, former results showed that the objective health status of teachers was better than that of the general population, especially regarding cardiovascular risk factors, e.g., obesity or dyslipidemia (28). One of the reasons for this difference might also be a better health behavior. School teachers showed better health behaviors compared to the general population. They were about half as likely to be smokers, more likely to exercise, and less likely to be overweight or underweight than the general population (28).

The most important predictor of self-rated health in our regression model was years of age, which is self-evident. Work-privacy conflicts and satisfaction with work also had a significant predictive value regarding self-rated health. Work-privacy conflicts can therefore be a factor to diminish the self-rated health status. As this is a cross-sectional study causalities can be reversed as well, as reduced self-rated health can influence work privacy conflicts, respectively, the appraisal of these conflicts. Another publication from the GHS-study with an overlapping sample to ours found a negative impact of work privacy conflicts on cardiovascular health, but only in women. Results for men were not significant (39).

#### 4.2.3. Satisfaction with life

Concerning satisfaction with life, the three occupational groups had mean values above the reference group of academics, i.e., they were on average more satisfied with their lives. School teachers and teaching and training professions were at a comparable level slightly below medical doctors and dentists, which showed the highest mean value. In the regression model, none of the occupational groups predicted satisfaction with life significantly. This result is surprising since other national and international studies (40) showed that life satisfaction of physicians can also be below that of the general population, respectively, of teachers (24). This study, which used the same scale for measurement, found slightly lower scores for teachers’ life satisfaction compared to our study. Differences are minimal though; the mentioned study was conducted in another German state so comparability might be affected by this.

In terms of sociodemographic variables we saw a clear positive association with socioeconomic status. Although our study already mostly included individuals with high socioeconomic status *per se*, higher socioeconomic status was significantly associated with higher life satisfaction. However, we did not see significant differences between male and female participants regarding their satisfaction with life. As it can be expected, satisfaction with work was a significant predictor for life satisfaction. This seems plausible, since work-related satisfaction is part of the more global life satisfaction. In addition, meaning of work was a further predictor beyond satisfaction with work, higher scores went along with more life satisfaction. Work-privacy conflicts on the other hand predicted lower life satisfaction.

### 4.3. Summary of job-related strains

In contrast to similar studies (23, 41), none of the considered occupational groups was a significant the predictor of job-related strains. Therefore, simply belonging to the occupational group of teachers was not significantly associated with higher levels of burnout, lower levels of self-rated general health, or lower levels of life satisfaction. It is possible that none of the occupational groups predicted the above outcome variables due to the high homogeneity between the groups. A Belgian study showed similar results to ours, differences in job demands and strains were only small when comparing teachers with other occupational groups (14).

Specific sociodemographic and job-related variables showed significant associations with the outcomes considered, but not the occupational group itself. Therefore, comparisons between occupational groups seem to fall short regarding the variables considered in the present study. Clear sex differences were found several times, especially with regard to higher burnout symptoms in women. Part-time employment, for example, was a significant risk factor for burnout only for female teachers, but not for males. This could be due to the fact that the backgrounds and motivations for taking up part-time employment might be different between the sexes. The fact that women in our study worked part-time more often than men could also be interpreted as an early attempt to reduce anticipated or existing multiple burdens, respectively, role expectations. Given these sex differences regarding burnout, it was unexpected to not find such differences in self-rated health as well as satisfaction. The higher scores for burnout in female teachers might partly be explained by the tendency that females tend to be more interested in inter- and intrapersonal processes than males (42) and is also in line with a role stereotypes perspective, from which it might seem to be more acceptable to admit mental burdens for females than for males.

Consistently across all three outcomes, work-privacy conflict was a significant predictor. Particularly in the case of school teachers, it seems worth taking a closer look at work-privacy conflicts, since part of the work takes place in the home environment and not only at the school workplace, which is systematically inherent in the profession of school teachers in general. An increased potential for a conflict between work and private life is accordingly expected to be found among school teachers. Corresponding results were reported in other studies with large sample sizes of school teachers where significantly higher values for work-privacy conflicts compared to the general population have been reported (24, 27). Teachers showed a 1.5 times higher level of work-privacy conflicts compared to a composition of other occupations (24).

Taken together these results, work-privacy conflicts seem to play a major role in teachers’ mental and somatic health and therefore should be one target for interventions to aim for.

### 4.4. Strengths and limitations

The exact classification of occupational groups according to the KLDB definition made it possible to clearly distinguish various occupational groups and to compare them with one another within the GHS data set. The fact that the participants live in the same region means that effects resulting from substantially different regional conditions can be excluded as far as possible. Yet, the external generalization of the results is limited because our sample is representative for the region of Mainz and Mainz-Bingen, Germany.

The job-related factors examined were derived from previous survey results, but other operationalizations could have led to different results. For example, variables like quantitative demands, the degree of freedom at work, workplace commitment, quality of leadership or social support could be additional, respectively, alternative ways to composite job-related factors.

Another limitation is the fact that the present study is cross-sectional, so that the variables cannot be interpreted in terms of causality. The division into predictors and outcome variables corresponds to the stress–strain concept (9) and the usual operationalization of other authors. However, it can be assumed that there is a reciprocal interaction, so that the assumed outcome variables (e.g., burnout) also influence the predictors (e.g., work-privacy conflict).

Furthermore, due to different sample sizes between the occupational groups, differences might not have been able to become significant. We did see some differences between the considered occupational groups, which were rather minimal.

A further limitation is that the data collected is based on subjective assessments and does not consider objective data (e.g., diagnosed disorders). However, this limitation applies to all occupational groups considered, so that at least no systematic bias due to the comparison of different data sources occurred between the different occupational groups. However, if there were systematic differences in the response tendencies of the corresponding occupational groups, this would be expressed here.

What can also be seen as a limitation to the study is, that we did not have data on non-responders, therefore corresponding analyses were not performed, which could have helped to get a clearer picture.

### 4.5. Outlook/implications

For further studies on the present topic, it would make sense to depict job-related factors more broadly than in the present study. Job-related factors, for example, could be operationalized by quantitative demands, social relationships at or connectedness with the workplace. The same applies to strains, for example these could be: intention to quit, clinical indicators (e.g., depression) or somatic symptoms. Another implication of the present study arises from the exclusive use of quantitative data. Particularly with regard to the sex-specific differences in the results, qualitative data, e.g., from individual interviews, could be a valuable supplement for future studies in order to gain insights into the everyday reality of part-time teachers, for example.

The high relevance of work-privacy conflicts with regard to all outcomes (burnout, self-rated health status, life satisfaction) is noteworthy. Other studies also identified work-privacy conflicts as a relevant predictor for certain strains (24, 27, 39). Interventions addressing this issue therefore could have multiple positive effects for teachers. Preventive or interventive strategies on behavioral level could be in form of trainings (e.g., face-to-face trainings or E-learnings) to establish a better separation between work and private life.

Trainings on how to deal with work-privacy conflicts should also highlight the chances that go along with structural conditions of the teachers’ job. High flexibility and compatibility with private life are positive aspects of the teachers’ profession. A reflection of these positive aspects could contribute to reframe teachers’ attitudes towards their job. Highlighting positive aspects of the teaching profession could also be beneficial for the professional socialization of teachers in terms of health.

Further useful interventions can be seen (1) on school level. Regulations regarding reachability for students and their parents or the allocation of working space in order to give support in the separation of work and private life could be helpful. (2) On a political level, expanding high-quality support systems (e.g., all-day childcare) would ease the burden on teachers who are parents. It would strengthen resources to cope with multiple stresses in order to improve the situation, for especially predominantly female teachers. Also, individual differences in the phase of life – e.g., career launch, parenting with differences for instance in terms of children’s age, nursing relatives – concern various needs of the employees. Those needs should be considered, on personal, school and political level, respectively.

Based on the available data, we cannot answer the question why females do not seem to benefit from a reduction in working hours with certainty, but we can reasonably assume that a gender role model that strives more toward equality – especially when it comes to childcare – could reduce existing inequalities. In order to be able to better grasp and deal with definite everyday problems that arise among school teachers with strongly pronounced work-privacy conflicts, in-depth studies addressing these issues are indispensable.

## Data availability statement

The data that support the findings of this study are available from the management board of the Gutenberg Health Study (GHS) but restrictions apply to the availability of these data, which were used under license for the current study, and so are not publicly available. Data are however available from the authors upon reasonable request and with permission of GHS.

## Ethics statement

All participants gave their written informed consent to participation. The Medical Ethics Commission of Rhineland‑Palatinate and local and Gutenberg‑University of Mainz data protection officials reviewed and approved of the study (ethics committee review number 837.020.07(5555)).

## Author contributions

TB and CK: conceptualization, methodology, formal analysis, writing – original draft, writing – review and editing, and visualization. PW and TM: validation, investigation, resources, writing – review and editing, supervision, project administration, and funding acquisition. MB: validation, investigation, resources, writing – review and editing, and supervision. KL: validation, investigation, resources, writing – review & editing, supervision, and funding acquisition. NP: validation, resources, review and editing, supervision, and funding acquisition. MN and SL: validation, writing – review and editing, supervision, and funding acquisition. JB: validation, writing – review and editing, and supervision. All authors contributed to the article and approved the submitted version.

## Funding

The Gutenberg Health Study is funded through the Government of Rhineland-Palatinate (“Stiftung Rheinland-Pfalz für Innovation”, contract AZ 961–386,261/733), the research programs “Wissen schafft Zukunft” and “Center for Translational Vascular Biology (CTVB)” of the Johannes Gutenberg-University of Mainz, and its contract with Boehringer Ingelheim and PHILIPS Medical Systems, including an unrestricted grant for the Gutenberg Health Study.

## Conflict of interest

The authors declare that the research was conducted in the absence of any commercial or financial relationships that could be construed as a potential conflict of interest.

## Publisher’s note

All claims expressed in this article are solely those of the authors and do not necessarily represent those of their affiliated organizations, or those of the publisher, the editors and the reviewers. Any product that may be evaluated in this article, or claim that may be made by its manufacturer, is not guaranteed or endorsed by the publisher.

## Footnotes

^1^SES was calculated as an index score (0–18) including the following variables: school education, professional education, occupational position, and salary.
